# P-1730. Fungi under fire: addressing a peruvian health challenge

**DOI:** 10.1093/ofid/ofaf695.1901

**Published:** 2026-01-11

**Authors:** Jon Salmanton-Garcia, Julio Maquera-Afaray, luis e cuellar, Oliver A Cornely

**Affiliations:** University Hospital Cologne, Cologne, Germany, Cologne, Nordrhein-Westfalen, Germany; Instituto de Investigación en Ciencias Biomédicas, Universidad Ricardo Palma, Lima, Perú, Lima, Lima, Peru; Instituto Nacional de Enfermedades Neoplásicas, Lima, Peru, lima, Lima, Peru; University of Cologne, Faculty of Medicine and University Hospital Cologne, Cologne, Nordrhein-Westfalen, Germany

## Abstract

**Background:**

Invasive fungal infections (IFI) are a significant global health issue, affecting an estimated 7 million people annually, with around 3 million deaths. In Peru, IFI are estimated to affect about 2% of the population. Underdiagnosis due to limited sensitivity of diagnostic tests underestimates the true burden. This study evaluates the diagnostic capabilities of mycology laboratories and the availability of antifungal treatments in Peruvian healthcare facilities to identify gaps and improve IFI management.

**Methods:**

An observational, cross-sectional study was conducted online targeting physicians involved in IFI management across multiple centres in Peru, from April 2023 to April 2024. The survey covered institutional profiles, incidence and perceived relevance of IFI, diagnostic tools, and access to antifungal drugs.

**Results:**

Fifty-four centres from 21/24 departments (Peruvian term for regions) in Peru participated. All centres reported a low to moderate IFI incidence. *Candida* spp. was the most concerning pathogen (93%), followed by *Aspergillus* spp. and *Cryptococcus* spp. (57% each). Diagnostic methods like microscopy were universally used (100%), while culture-based diagnosis was available in 90% of centres. Access to advanced diagnostics for species identification varied, with better availability in the capital (91%) compared to regions (64%). Antibody detection tests were available in 30% of centres, mostly in the capital area. Antigen detection tests were available in 46% of institutions, with significant regional disparities. Imaging techniques were widely used, but surgical access varied. Triazoles were the most accessible antifungals (96%), while echinocandins and therapeutic drug monitoring (TDM) were significantly limited (37% and 2%, respectively).

**Conclusion:**

The study highlights disparities in the availability of advanced diagnostics and antifungals in Peru. Despite universal use of microscopy, access to species identification, antibody, and antigen detection tests is limited outside the capital. Ensuring equitable access to these resources and implementing therapeutic drug monitoring are crucial for improving IFI management in Peru.

**Disclosures:**

Jon Salmanton-Garcia, MSc, MPH, PhD, menarini, gilead, astrazeneca, pfizer: Honoraria Oliver A. Cornely, Prof. Dr., Al-Jazeera Pharmaceuticals/Hikma: Honoraria|Basilea: Advisor/Consultant|Cidara: Advisor/Consultant|Cidara: Board Member|Cidara: Grant/Research Support|Elion: Advisor/Consultant|F2G: Grant/Research Support|Gilead: Advisor/Consultant|Gilead: Grant/Research Support|Gilead: Honoraria|GlaxoSmithKline: Advisor/Consultant|GlaxoSmithKline: Honoraria|Grupo Biotoscana/United Medical/Knight: Honoraria|Melinta: Advisor/Consultant|Melinta: Board Member|MSD: Honoraria|Mundipharma: Advisor/Consultant|Mundipharma: Grant/Research Support|Mundipharma: Honoraria|Pfizer: Advisor/Consultant|Pfizer: Grant/Research Support|Pfizer: Honoraria|Pulmocide: Board Member|Scynexis: Advisor/Consultant|Scynexis: Grant/Research Support|Shionogi: Advisor/Consultant|Shionogi: Honoraria

